# Risk-based prioritization of operation and maintenance indicators for school sanitation decision support in Kampala City, Uganda

**DOI:** 10.1007/s43832-026-00412-4

**Published:** 2026-06-11

**Authors:** Jude Zziwa Byansi, Swaib Semiyaga, Alex Yasoni Katukiza, Najib Lukooya Bateganya, Angella Mercy Nakaye, Frank Kansiime, Robinah Nakawunde Kulabako

**Affiliations:** 1 Department of Civil and Environmental Engineering, College of Engineering, Design, Art and Technology, Makerere University, P.O. Box 7062, Kampala, Uganda; 2 Department of Environmental Management, College of Agricultural and Environmental Sciences, Makerere University, P.O. Box 7062, Kampala, Uganda; 3 Benign Gynecology Department, Mulago Specialized Women and Neonatal Hospital, P.O. Box 2208, Kampala, Uganda

**Keywords:** School sanitation, Operation and maintenance, Risk-based prioritization

## Abstract

Effective operation and maintenance (O&M) of sanitation facilities are vital for ensuring consistent service delivery in schools, yet many low- and middle-income contexts continue to face challenges stemming from reactive maintenance practices and weak management systems. This study developed and applied a risk-based approach to identify and prioritize O&M processes that most threaten the continuity of sanitation services in Kampala City schools. Using a cross-sectional workshop-based design, 106 head teachers and 99 sanitation teachers from 205 schools assessed the status, likelihood, and severity of failure for 33 indicators across six O&M domains. Risk scores were computed following the adapted World Health Organization’s Sanitation Safety Planning framework, and indicators were ranked using median (P50) and upper-quartile (P75) statistics to identify priority areas. The results showed that 46% of the indicators were rated as low risk, 24% as medium risk, and 30% as high risk, with no indicator classified as very high-risk. Material supply and use management, toilet cleanliness, preventive maintenance schedules, menstrual hygiene material provision, and sanitation budget-line implementation were identified as the most critical areas requiring attention. Although several monitoring and evaluation indicators ranked as lower risk, this reflects their strong current performance rather than a reduced importance in sustaining sanitation services. The findings highlight that the main threats to sanitation service reliability arise from weak O&M practices and resource management processes rather than infrastructure deficits. Targeting preventive maintenance, supply management, and budget execution provides a practical pathway for strengthening the functionality, reliability, and continuity of sanitation services within resource-limited school environments.

**Graphical abstract:**

**Figure:**
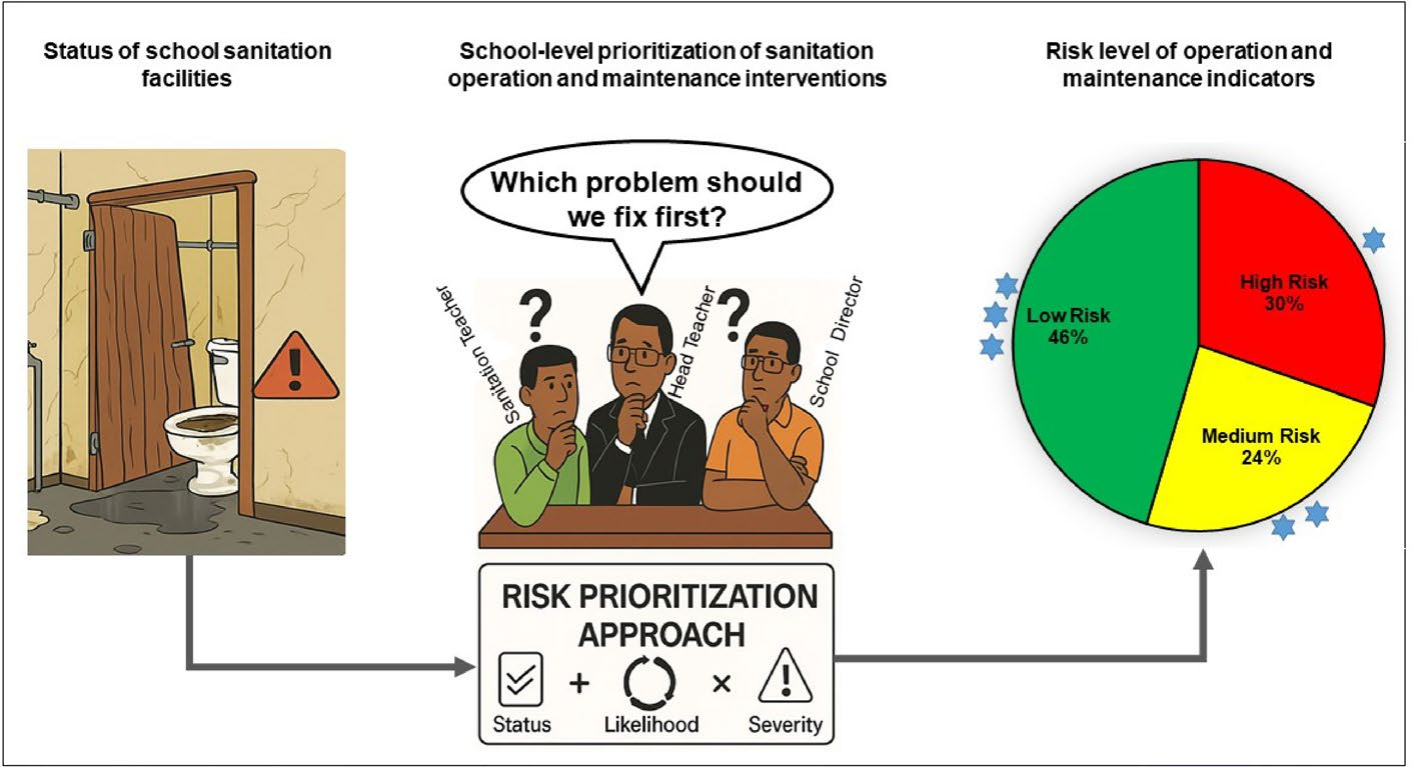

**Supplementary Information:**

The online version contains supplementary material available at 10.1007/s43832-026-00412-4.

## Highlights

- A risk-based approach prioritizes school sanitation O&M indicators in Kampala.
- Weak O&M practices pose greater risks than sanitation infrastructure deficits.
- Supply management, cleanliness, and preventive maintenance rank highest risk.
- SSP-based scoring enables evidence-driven school-level sanitation decisions.
- Findings support practical DSS development for sanitation O&M in schools.

**Supplementary Information:**

The online version contains supplementary material available at 10.1007/s43832-026-00412-4.

## Introduction

Sustained sanitation service delivery extends beyond infrastructure provision to include management systems that ensure functionality, reliability, and quality of services [42]. Evidence shows that improved sanitation facilities do not always deliver basic service levels when operation and maintenance (O&M) practices such as water availability, cleaning, and pit emptying are inadequate [27]. Improved sanitation facilities are those that hygienically separate human excreta from human contact, including flush or pour flush systems, ventilated improved pit latrines, pit latrines with slabs, and composting toilets. However, achieving a basic sanitation service level requires that such facilities are available, functional, sex segregated, and provide privacy. In many school settings, routine cleaning, preventive maintenance, supply management, and monitoring mechanisms are inadequately implemented or inconsistently applied resulting in recurrent service failures despite the availability of improved facilities [21]. Globally, an estimated 205 million pupils use improved sanitation facilities that do not meet basic service level due to management-related factors such as inadequate sex segregation and poor facility usability [41]. This situation reflects not only infrastructure gaps but also weaknesses in the systems responsible for operation and maintenance (O&M), which must sustain the functionality, cleanliness, and inclusiveness of school sanitation facilities [11, 42]. These challenges are more pronounced in high-use, multi-user settings, where maintenance demands are greater and service quality is more difficult to sustain, particularly under seasonal variations in water availability and facility use [22, 26].

Operation and maintenance encompasses all activities required to keep sanitation systems functional and hygienic, including daily use, cleaning, inspection, repair, budgeting, and supervision [29]. The effectiveness of these O&M processes is also influenced by school leadership and community behavior, which shape accountability and facility use, while reliable water availability enables routine cleaning and hygiene practices necessary to sustain service levels [8, 13, 16]. Inadequate O&M frequently results in non-functional or dirty toilets, water leakages, and unreliable water supply, all of which increase pupils’ exposure to sanitation-related diseases and adversely affect health, attendance, and learning outcomes [8]. These failures do not occur suddenly but emerge progressively through missed inspections, delayed repairs, and unnoticed defects. Interruptions in sanitation service provision often follow predictable patterns and present early warning signs, allowing them to be anticipated and prevented when risks are systematically identified. To address these challenges, a preventive, risk-based management approach is required.

Risk is defined as the combined function of the likelihood of an adverse event and the severity of its consequences [4, 42]. Managing risks within sanitation systems requires systematic monitoring of O&M processes, interpretation of indicator data, and the use of this evidence to guide timely service improvements. The state of a sanitation system is best understood through measurable indicators: quantitative or qualitative signs that reflect the performance of processes or conditions influencing service delivery [8, 40]. Routine monitoring of these indicators enables managers to detect emerging problems, take corrective action before failures escalate, and strengthen the reliability of school sanitation services. Reducing the probability of indicator failure consequently lowers pupils’ exposure to O&M-related health and hygiene risks.

Evidence from low- and middle-income countries (LMICs) shows persistent problems such as non-functional toilets, lack of gender-segregated facilities, absence of soap or water, and poor cleanliness [2, 5, 9, 16]. These challenges can be attributed to weak O&M practices and the absence of systematic tools for prioritizing maintenance needs [20, 42]. Existing decision-support tools, such as Sanitation Safety Planning (SSP) framework, the excreta flow diagram (SFD), the Citywide Inclusive Sanitation—Service Assessment and Planning (CWIS-SAP) tool, and the Knowledge-to-Practice (K2P) tool, have improved risk-based sanitation planning at municipal and utility levels [14, 17, 31, 36, 42]. However, they are data-intensive, and not adapted to the school context, where decisions are often made by non-technical personnel with limited resources. As a result, it remains unclear which sanitation indicators pose the greatest risks to service continuity and pupil well-being, hindering effective prioritization during corrective processes.

The objective of this study was to develop an approach that supports school-level actors in identifying and prioritizing O&M indicators representing critical service processes for targeted intervention to improve sanitation service delivery in schools. The approach aims to enable non-technical personnel to make informed, evidence-based decisions that strengthen the effectiveness and sustainability of sanitation service provision. The findings provide a practical method for prioritizing O&M indicators in resource-constrained school settings and contribute empirical evidence to guide risk-based decision-making for improved routine maintenance at the school level.

## Study methodology

### Study area

The study was conducted in Kampala, the capital and largest city of Uganda, located at 0°19′ N, 32°35′ E, about 10 km north of Lake Victoria and 45 km from the Equator [25]. The city has roughly 1.88 million residents, of whom over 85 percent rely on onsite sanitation systems such as pit latrines and septic tanks [23, 37]. Kampala hosts about 683 primary and 169 secondary schools, with 88 percent privately owned and the rest being public schools [8]. Most schools lack structured monitoring mechanisms, and sanitation data are mainly collected through paper-based inspection reports, making Kampala a representative setting for assessing urban school sanitation management.

### Study design

This study adopted a cross-sectional, workshop-based design to assess O&M performance and associated risks in school sanitation systems using a structured indicator-based approach. A cross-sectional design was considered appropriate because the study aimed to obtain a snapshot assessment of the current status, likelihood of failure, and severity of consequences associated with sanitation O&M processes across schools at a single point in time. The design enabled comparison of risk conditions across a large number of schools within the available time and resource constraints.

The study used a set of 33 O&M sanitation indicators in school settings that had been identified and refined during an earlier phase of the wider research project through a structured review of the literature and expert consultations (Byansi et al. n.d.). The selected indicators were intended to operationalize and evaluate key O&M processes within school sanitation systems, forming the core analytical focus of this study. The indicators were organized into six domains representing major O&M processes across the sanitation service chain, including service planning, compliance with facility design standards, resource allocation and utilization, service availability, governance, and monitoring and evaluation to support quality sanitation service provision (Supplementary material S1 & S2).

The organization of the indicators was guided by project life-cycle processes and the Joint Monitoring Programme sanitation service-level frameworks. However, results are presented at the individual indicator level to preserve variability and support targeted prioritization of specific O&M processes. In the present study, the 33 indicators were applied to assess their status, likelihood of failure, and severity of consequences in order to determine priority areas for strengthening operation and maintenance in schools.

The required sample size was estimated using the Cochran formula for categorical data and adjusted for a finite population of 852 schools in Kampala City (N < 10,000), margin of error of 5% and estimated proportion of 50% for maximum sample size. A final sample size of 265 schools, comprising 211 primary and 54 secondary schools, including 33 public and 232 private institutions, was selected through proportionate stratified random sampling based on administrative division, ownership, and education level to ensure representativeness. Each selected school was invited to nominate either a Head Teacher or a Sanitation Teacher as a participant, maintaining a balance between school administrators and implementers. Head Teachers and Sanitation Teachers were selected because they are directly involved in school sanitation planning, supervision, budgeting, and daily facility management, and therefore possess contextual knowledge on the functionality and implementation of O&M processes within schools.

A structured questionnaire was administered to capture for each indicator: (i) the status of implementation, (ii) the likelihood of failure or breakdown, and (iii) the severity of consequences should that failure occur. The questionnaire consisted of standardized items requiring participants to evaluate indicator status based on their knowledge of school conditions and available records, while likelihood and severity ratings were based on their professional judgement and experience. The questionnaire-based approach was considered appropriate because several O&M processes, including maintenance scheduling, supervision, supply management, and budgeting, are institutionally managed and could not be adequately assessed through one-time facility observations alone. The use of standardized questionnaire items therefore enabled consistent assessment of implementation status and associated risks across a large number of schools. The questionnaire was adapted from the Sanitation Safety Planning (SSP) framework assessment procedures.

To ensure consistency in responses, participants attended a two-hour training session on risk assessment principles and the World Health Organization (WHO) Sanitation Safety Planning (SSP) framework, with emphasis on standardizing interpretation of likelihood and severity ratings [42]. The training was intended to minimize variability in interpretation of risk concepts and improve scoring consistency across participants. The full questionnaire, including indicator definitions and scoring criteria, is provided in Supplementary material S2.

A total of five workshops were conducted, one in each of the five administrative divisions of Kampala City. Each workshop was facilitated by one lead researcher and four trained Research Assistants (RAs) who guided participants through the questionnaire and verified completeness before submission. This approach enabled a structured, expert-informed assessment of O&M performance and risk conditions within school sanitation systems.

The workshop method was adopted because it provided a participatory environment for engaging practitioners with direct management and operational experience in school sanitation. It enabled collective reflection, standardized interpretation of risk concepts, and ensured that responses reflected institutional realities rather than individual opinions. The workshop setting also improved uniformity in administration of the questionnaire and reduced inconsistencies that could arise from independent self-administration of risk assessment tools. Moreover, convening participants in Divisional clusters minimized data-collection costs and promoted peer verification of school-level assessments, enhancing both the quality and validity of the data collected.

### Data processing and analysis

Data from the completed questionnaires were entered in comma-separated values (CSV) format and imported into Python (version 3.12) for cleaning, validation, and analysis. The dataset was examined for completeness, missing entries, logical consistency, and outliers. Because all variables were ordinal, derived from Likert-type responses, no continuous data were subjected to normality tests. The data were summarized using descriptive and non-parametric statistics. Non-parametric approaches were applied because the ordinal data did not satisfy key parametric assumptions, including normal distribution, homogeneity of variances, and equal interval spacing between response categories.

Each sanitation indicator consisted of three components: status, likelihood, and severity (Supplementary Material 2). The status of an indicator was rated on a five-point scale where one represented an absent or non-existent process and five denoted a fully functional or fully implemented process. Likelihood was rated on a five-point scale ranging from one for very unlikely to five for almost certain. Severity followed a non-uniform five-level scale in which one represented an insignificant, two for minor, four for moderate, eight for major, and sixteen for catastrophic consequences as adapted from the SSP framework.

Following the SSP framework, a semi-quantitative risk score was computed for each indicator as the product of likelihood and severity [42]. This semi-quantitative approach enabled simultaneous consideration of both the probability of failure and the magnitude of associated consequences during prioritization of sanitation O&M indicators. The resulting scores were graded according to the SSP risk matrix, where values below six were categorized as low risk, those between six and twelve as medium risk, those between thirteen and thirty-two as high risk, and those above thirty-two as very high risk (Supplementary material 3).

The median (P50) of the risk scores for each indicator was computed to represent the central tendency of perceived risk among participants and was considered appropriate for ordinal and non-normally distributed data. Indicators were subsequently ranked in descending order of their median values, with the upper quartile (P75) applied as a secondary ranking criterion and the interquartile range (IQR) incorporated to reflect the level of consensus among participants. Indicators exhibiting smaller IQR values demonstrated higher agreement and greater reliability in ranking, whereas those with wider IQRs reflected higher variability in expert judgment. This integrated procedure established a systematic and transparent approach for identifying indicators that were most consistently perceived as high risk.

The internal consistency of the questionnaire constructs was verified using Cronbach’s alpha, and Spearman’s rank correlation applied to examine associations among the three risk components (status, likelihood, and severity).

## Results and discussion

### Workshop participation and respondent characteristics

Out of the 265 participants invited to attend the five calibration workshops, 205 (77%) participated in the study (Table 1). The participants comprised head teachers and sanitation teachers from both primary (77%) and secondary (23%) schools, with an average of 15 years of work experience. Most of the participating schools were privately owned (75%) and mainly primary-level institutions (77%). Slightly more than half of the schools met the recommended pupil-to-stance ratio of 40:1, while the remainder exceeded this standard.

**Table 1:** Participant and school characteristics (N = 205)

Participant characteristics	Number (n)	Percentage (%)
Male	82	40.0
Female	123	60.0
Head Teacher	106	51.7
Sanitation Teacher	99	48.3
Average experience (years)	15	
*School characteristics*		
Public school	52	25.4
Private school	153	74.6
Primary	157	76.6
Secondary	48	23.4
Meeting PSR of 40:1	120	58.5
Above PSR of 40:1	85	41.5

The 77% participation rate in the workshops was influenced by the timing of data collection, which coincided with the national examination period. During this time, teachers are typically engaged in examination preparation, training, and invigilation activities organized by the examination body, limiting schools’ ability to release staff for external engagements. The near-equal representation of head teachers and sanitation teachers ensured inclusion of both administrative and operational perspectives in the assessment. Participants’ average professional experience of 15 years indicates long-term involvement in school management and sanitation practices. The finding that 42% of schools did not meet the national pupil-to-stance ratio (PSR) standard of 40:1 is consistent with the results of [7–9], who reported that 44% of Kampala schools were below the same benchmark, confirming persistent inadequacies in sanitation infrastructure across the city’s schools. High pupil-to-stance ratios above the national standards are also widely reported in studies in developing countries underscoring how crowding at toilets remains a common constraint to service quality [10, 18, 24].

### Status levels of operation and maintenance indicators for school sanitation facilities

**Fig. 1:**
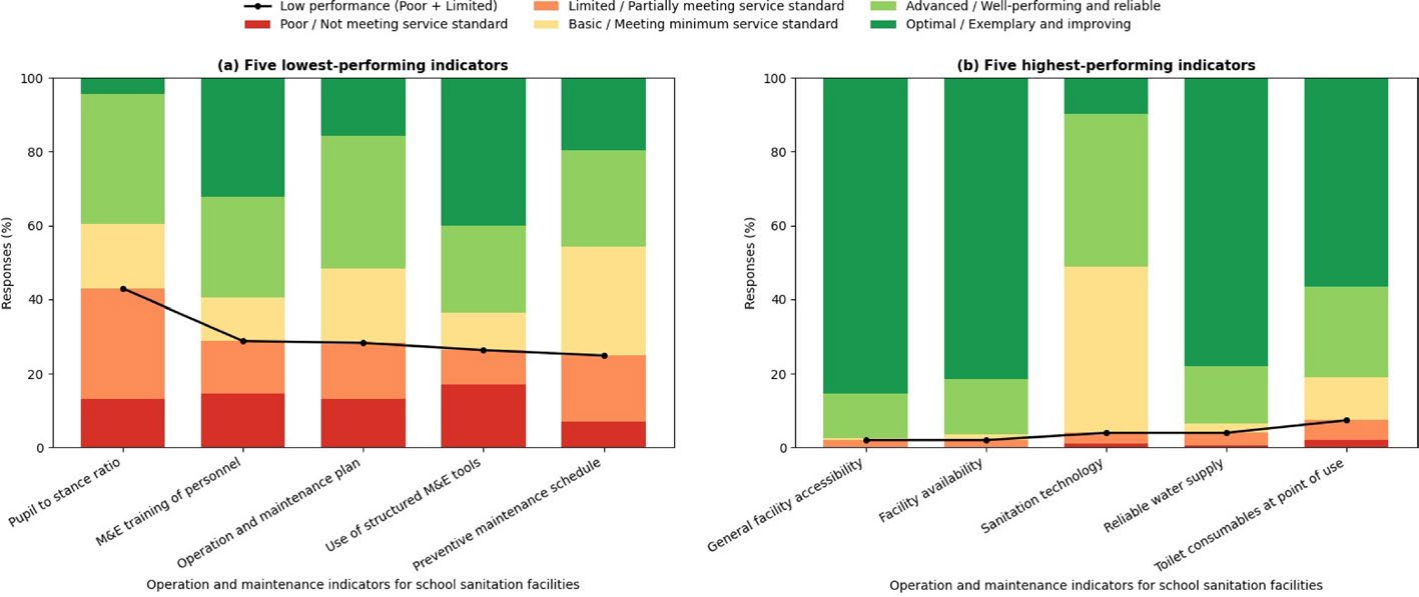
Distribution of status levels for selected operation and maintenance indicators across schools, ranked by the combined proportion of Poor and Limited responses: **a** five lowest-performing indicators and **b** five highest-performing indicators

The self-reported status of O&M indicators varied across schools (Fig. 1 and Supplementary material 4), with indicators arranged in descending order of the combined proportion of Poor and Limited performance levels. These findings are based on expert-informed assessments and should be interpreted in that context. The pupil-to-stance ratio showed the highest proportion of schools at Poor and Limited performance levels (43%). Monitoring and evaluation-related indicators showed similarly high proportions of Poor and Limited performance (25–29% of schools), including training of personnel, use of structured monitoring tools, and escalation of unresolved issues. In contrast, indicators associated with facility-level conditions, such as facility accessibility, facility availability, water supply reliability, sanitation technology, and the presence of consumables at the point of use, showed low proportions of Poor and Limited performance (3–7% of schools), with most schools falling within the Basic, Advanced, and Optimal performance levels.

The high proportion of schools not meeting the recommended pupil-to-stance ratio observed in this study reflects a persistent challenge reported in school sanitation literature in LMICs, where schools frequently lack sufficient toilet facilities and adequate sanitation infrastructure to meet user demand [7, 28, 30, 39]. This is often driven by increasing school enrolment without a corresponding expansion of sanitation facilities. Addressing this gap requires targeted investment in sanitation infrastructure, including construction of additional toilet stances, rehabilitation of non-functional facilities, and maintenance of existing infrastructure to meet recommended standards and reduce user pressure.

The relatively poor performance of monitoring and evaluation-related indicators, including training of personnel, use of structured monitoring tools, and escalation of unresolved issues, highlights critical gaps in institutional O&M processes. Similar findings have been reported by Duijster et al. [16], who showed that weak monitoring and management systems limit the effectiveness of sanitation services even where facilities exist. Evidence from school WASH studies further indicates that sanitation interventions are often inconsistently implemented and difficult to sustain over time, reflecting gaps in routine system support and implementation processes [15, 19]. These findings also point to the importance of institutional and management conditions in shaping O&M performance, as organizational and contextual factors influence the maintenance and use of sanitation facilities [10]. Strengthening these processes through capacity building, adoption of structured monitoring tools, and establishment of clear reporting and response mechanisms is therefore essential for improving the sustainability of sanitation services.

In contrast, indicators associated with facility-level conditions, such as accessibility, availability, water supply reliability, sanitation technology, and the presence of consumables, showed relatively better performance. This suggests that while basic sanitation infrastructure may be in place, maintaining service quality requires effective operation and maintenance systems [42], as service levels depend on the functionality, availability, and usability of facilities (WHO/UNICEF 2022). Overall, the findings indicate that improving sanitation service delivery requires a combined approach that prioritizes both expansion of infrastructure and strengthening of monitoring and management systems.

### Relationships among status, likelihood, and severity ratings for the indicators

**Fig. 2:**
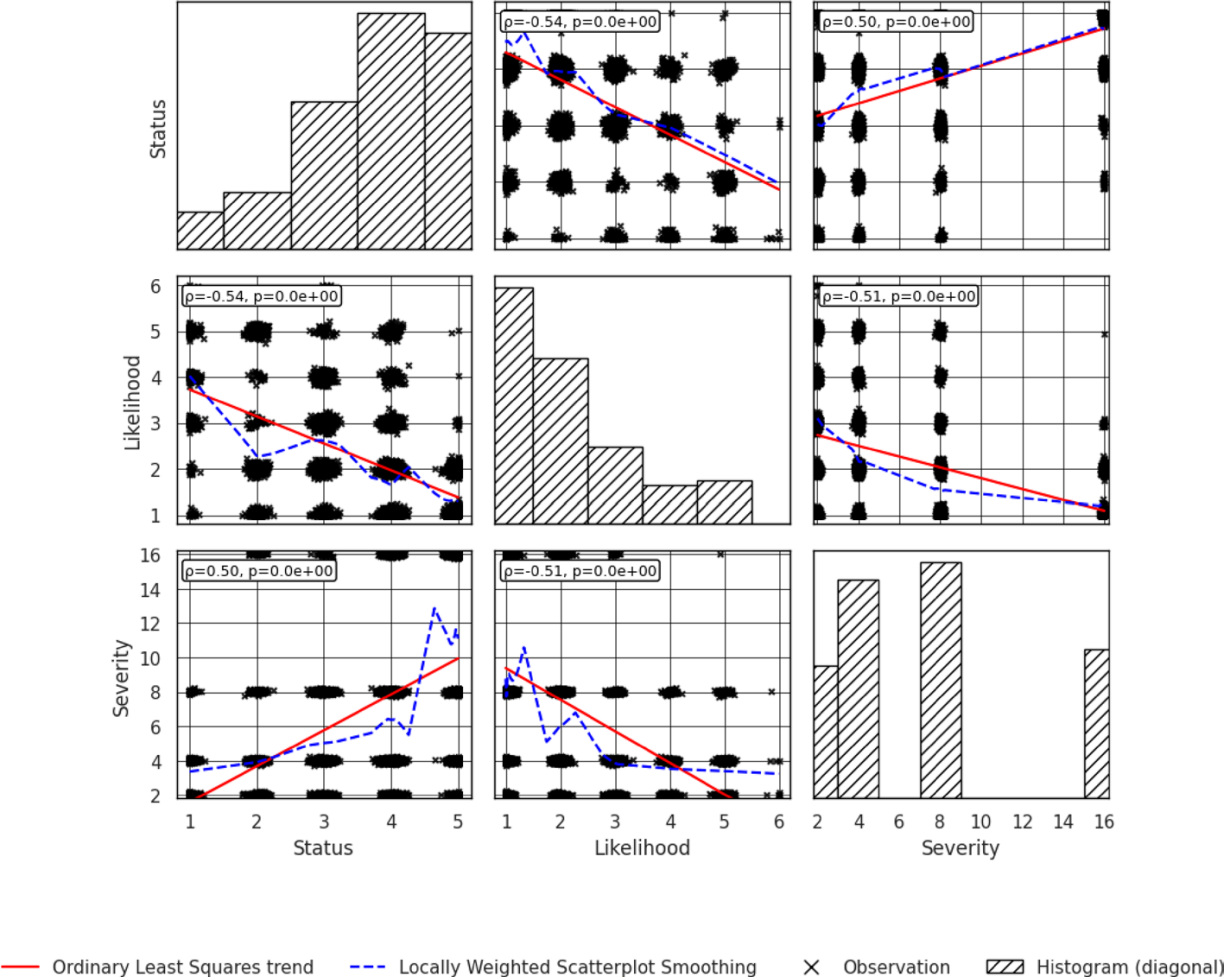
Associations among status, likelihood, and severity ratings of operation and maintenance indicators

A moderate negative correlation (ρ = − 0.54, p < 0.001) was observed between status of O&M indicators and the likelihood of their failure, while a moderate positive correlation (ρ = 0.50, p < 0.001) was recorded between status and severity of consequences with such failure (Fig. 2). In this study, likelihood refers to the probability that an O&M process or condition represented by a given indicator may fail or be inadequately implemented within the school sanitation system, while severity refers to the magnitude of the resulting consequences of such failure or inadequate implementation on sanitation service delivery [3, 42]. Examples of such failures include unavailability of sanitation facilities for use, interruptions in material supply systems, failure to maintain reliable water supply, failure to place toilet consumables at the point of use, and unsafe containment of excreta. Likelihood and severity scores also exhibited a negative association (ρ = –0.51, p < 0.001). The diagonal histograms show that most indicators were concentrated around higher performance levels and lower likelihood scores, with greater variability observed in severity ratings.

The observed moderate negative correlation between status and likelihood (ρ = − 0.54, p < 0.001) indicates that indicators with higher functional status are perceived as less likely to fail. Schools operating at higher status levels tend to have systems that reduce the likelihood of failure, whereas schools at lower status levels experience conditions that increase the probability of failure. This finding aligns with studies showing that stronger O&M systems are associated with fewer service disruptions in school sanitation [6, 12, 16].

The significant positive relationship between status and severity (ρ = 0.50, p < 0.001) indicates that schools with higher operational status perceive failures as having more serious implications, whereas those accustomed to lower service quality tend to view such failures as less consequential. Schools maintaining high sanitation standards typically engage actively in risk management, interpreting failures as opportunities for system improvement and taking preventive measures to mitigate risks. Conversely, institutions with a persistent history of inadequate service provision often exhibit low levels of preparedness and limited engagement with proactive risk management, contributing to organizational environments where failures are insufficiently anticipated or systematically addressed [34]. The negative correlation between severity and likelihood (ρ = − 0.51, p < 0.001) reflects a common risk pattern where frequent failures are low in impact, while rare failures carry higher consequences [38, 43]. To prevent catastrophic outcomes, schools often establish mechanisms that safeguard high-severity indicators, resulting in lower likelihood scores, while dedicating less effort to failures with minimal consequences.

These relationships highlight the importance of maintaining high functional status to minimize the likelihood of failure and of giving sustained attention to high-severity domains even when their likelihood is low. School sanitation programs should therefore allocate resources strategically, prioritizing routine maintenance to prevent recurrent failures and strengthening monitoring and contingency systems to detect and mitigate high-consequence events.

### Intervention prioritization of operation and maintenance indicators of sanitation facilities across schools

Nearly half (46%) of the operation and maintenance indicators were rated as low risk, while 30% were categorized as high risk, with none reaching the very high-risk level. Apart from the material supply and toilet cleanliness indicators, all other processes had a relatively narrow interquartile range of eight units, indicating limited variability across schools. Material supply and use management emerged as the highest-ranked indicator for prioritization, followed by toilet cleanliness (Table 2). A cluster of eight indicators occupied the third rank, four of which were menstrual hygiene material availability, toilet facility accessibility, facility availability, and facility functionality, were linked to service delivery. Conversely, the monitoring and evaluation of personnel was ranked lowest, followed by a group of eleven indicators occupying the twenty-second rank. Rank 22 was dominated by facility design standards, including privacy, menstrual hygiene facility provision, sanitation technology, sex segregation, and the presence of handwashing facilities near toilets. Among the five indicators under the monitoring and evaluation theme, three were rated low risk and ranked 22.

**Table 2:** Median risk levels (P50) and quartile statistics for intervention prioritization of operation and maintenance indicators of school sanitation facilities

Indicator name	Median (50th percentile)	Risk level	Upper quartile	Inter quartile range	Rank (tie)
Material supply and use management	16	High	32	20	1
Toilet cleanliness	16	High	20	12	2
Preventive maintenance schedule	16	High	16	8	3
MHM materials availability	16	High	16	8	3
Budget-line implementation	16	High	16	8	3
Reliable water supply	16	High	16	8	3
Facility availability	16	High	16	8	3
Facility accessibility	16	High	16	8	3
Facility functionality	16	High	16	8	3
Safe excreta disposal	16	High	16	8	3
Facility accessibility by the people with disabilities (PWDs)	12	Medium	16	8	11
Operation and maintenance plan	12	Medium	16	8	11
Roles and responsibilities	12	Medium	16	8	11
Dedicated budget-line for O&M	12	Medium	16	8	11
Safe containment of excreta	12	Medium	16	8	11
Escalation of unresolved issues	12	Medium	16	8	11
Toilet consumables at point of use	12	Medium	16	8	11
Rules and regulation enforcement	12	Medium	16	8	11
Pupil to stance ratio	10	Low	16	8	19
Trained personnel	10	Low	16	8	19
Formal management structures	10	Low	16	8	19
Multiple stakeholder monitoring	8	Low	16	8	22
Privacy	8	Low	16	8	22
MHM facility provision	8	Low	16	8	22
Stakeholder engagement	8	Low	16	8	22
Sanitation technology	8	Low	16	8	22
Sex segregation	8	Low	16	8	22
Handwashing facilities	8	Low	16	8	22
Safe disposal of solid waste	8	Low	16	8	22
Hygiene promotion activities	8	Low	16	8	22
Use of structured M&E tools	8	Low	16	8	22
Monitoring data use	8	Low	16	8	22
M&E training of personnel	8	Low	12	8	33

The ranking of O&M indicators revealed that processes related to material supply and use management, toilet cleanliness, preventive maintenance schedules, and menstrual hygiene material availability represent the highest risk indicators in Kampala schools. This finding is consistent with Buxton et al. [6] and Duijster et al. [16] who reported that inadequate supply systems and irregular cleaning routines are major causes of sanitation service failure in low-resource educational settings. The high risk attributed to these indicators reflects the fragile nature of supply chains and the reactive approach to maintenance in many sub-Saharan institutions, where limited budgets and weak procurement systems restrict routine replenishment and repair. Comparable findings were reported by Tumwebaze [35] who observed that neglecting O&M practices increases failure likelihood for sanitation systems. Strengthening supply management mechanisms, establishing structured cleaning schedules, and integrating preventive maintenance into school routines are essential to reduce these risks.

A second cluster of high-risk indicators, including budget-line implementation, reliable water supply, facility availability, accessibility, and functionality, demonstrates that operational constraints extend beyond consumables to structural and financial limitations. Inadequate financing and unreliable water supply undermine sustainability even where sanitation infrastructure is available [1, 33]. In Kampala public schools, inadequate funding and planning for sanitation were highlighted as major blockages to adequate sanitation [24]. Coordinated financial planning and the inclusion of emergency water storage systems could minimize these vulnerabilities.

Medium-risk indicators such as accessibility for pupils with disabilities, the presence of operational plans, and defined roles and responsibilities indicate partial institutionalization of O&M processes. The limited variation across these indicators suggests uniform but shallow compliance. Institutionalizing O&M activities in school performance assessments, clarifying accountability for sanitation focal teachers, and scheduling routine supervisory checks can strengthen adherence to planned practices.

Indicators currently rated as low risk, mainly from the facility-design domain such as privacy, sex segregation, sanitation technology, and handwashing facilities, represent comparatively lower rankings during prioritization. However, schools should remain vigilant to prevent regression in these areas. The finding of low risk for indicators like sanitation technology is consistent with [8, 9], who reported that all schools in Kampala used improved sanitation facilities. Evidence from school WASH studies shows that limited operational attention to well-performing areas can lead to gradual deterioration, particularly where monitoring and feedback structures are weak [15, 32]. This underscores the importance of embedding periodic sanitation audits and structured review mechanisms within school operations to ensure early identification of defects, maintain functionality, and reduce the likelihood of future service failures.

The risk-based prioritization methodology demonstrates that the most pressing vulnerabilities in school sanitation systems stem from operational and resource management deficiencies rather than infrastructure design. Prioritizing corrective action on supply management, cleaning, maintenance scheduling, and resource allocation should therefore form the foundation of school-level decision support and citywide O&M improvement strategies.

## Implications for policy, practice, and future research

The findings indicate that most sanitation risks in Kampala schools arise from operational and management weaknesses rather than infrastructural deficiencies. Policymakers should therefore reorient their focus from the provision of physical facilities toward strengthening systems that support continuous O&M of the facilities. Establishing dedicated and ring-fenced budget lines for sanitation O&M at both the school and city levels would ensure predictable financing for consumables, repairs, and preventive maintenance. Integrating risk-based monitoring within the Education Management Information System (EMIS) would further enhance evidence-driven planning, enable early detection of service failure, and facilitate targeted resource allocation across schools.

At the implementation level, the prioritization results provide a practical roadmap for improving sanitation performance through targeted interventions. Strengthening material supply systems, instituting structured cleaning schedules, and embedding preventive maintenance activities within school timetables should become standard operational practices. Training sanitation teachers in risk-based assessment and documentation would enhance responsiveness and accountability, while the adoption of simple checklists, feedback mechanisms, and routine inspections by health inspectors could institutionalize regular monitoring and sustain service functionality.

From a research perspective, this study provides the empirical foundation for developing a Decision Support System (DSS) that applies risk-based analytics to guide O&M management in schools. Future research should therefore focus on translating the identified high-risk indicators into real-time monitoring modules, testing the predictive accuracy of risk trends across different school contexts, and assessing the cost-effectiveness of risk-based interventions compared with routine maintenance approaches.

## Study limitations

This study applied a workshop-based cross-sectional design that relied on expert judgment rather than direct observation. The use of Likert-type scales introduced an element of subjectivity, and responses may have been influenced by differences in interpretation or institutional context. These limitations were mitigated through standardized training, alignment with established frameworks such as the World Health Organization’s Sanitation Safety Planning protocol, guided facilitation, and the use of non-parametric statistics appropriate for ordinal data.

## Conclusion

This study developed and applied a risk-based approach to identify and prioritize operation and maintenance (O&M) processes that pose the greatest threats to sanitation service continuity in Kampala schools. The analysis identified high risk O&M indicators including material supply management, preventive maintenance, menstrual hygiene material provision, and toilet cleanliness based on their combined status, likelihood of failure and severity of consequences. The findings suggest that sanitation service gaps in schools are associated more with weaknesses in maintenance systems, particularly in monitoring and resource management processes than with infrastructural limitations alone. Ranking O&M indicators according to their risk magnitude provides a structured basis for schools to allocate resources and direct interventions toward the processes most likely to disrupt sanitation service delivery. The approach offers a practical tool to support non-technical school personnel in making evidence-based maintenance decisions that improve the functionality, reliability, and long-term performance of sanitation facilities in resource-constrained environments.

## Supplementary Information

Below is the link to the electronic supplementary material.

Supplementary Material 1.

## Data Availability

Data are available at [10.5281/zenodo.18061933].

## Abbreviations

CSV: Comma separated values
DSS: Decision support system
EMIS: Education management information system
IQR: Inter quartile range
JMP: Joint monitoring programme
KCCA: Kampala Capital City Authority
M&E: Monitoring and evaluation
MHM: Menstrual hygiene management
O&M: Operation and maintenance
P50: Median value (50th percentile)
P75: Upper quartile (75th percentile)
PSR: Pupil to stance ratio
PWD: People with disabilities
RA: Research assistant
RAs: Research assistants
SDGs: Sustainable development goals
SSP: Sanitation safety planning
UBOS: Uganda Bureau of Statistics
UNCST: Uganda National Council for Science and Technology
UNICEF: United Nations Children’s Fund
WASH: Water sanitation and hygiene
WHO: World Health Organization
