# Supplementary material for: Risk-based prioritization of operation and maintenance indicators for school sanitation decision support in Kampala City, Uganda
Source: Discov Water. 2026 Jun 11;6(1):89. doi: 10.1007/s43832-026-00412-4 (PMC13481572; doi:10.1007/s43832-026-00412-4)
Supplement: Supplementary file 1 [file 43832_2026_412_MOESM1_ESM.docx]

**Supplementary S1: School sanitation facilities operation and maintenance domains and indicators (Adopted from** (Byansi et al. n.d.)**)**

| **School sanitation facilities operation and maintenance Domain** | **School sanitation facilities operation and maintenance Indicator** |
| --- | --- |
| Service planning | O&M planning |
|  | Clear roles and responsibilities |
|  | Dedicated budget-line |
|  | Preventive maintenance schedule |
| Facility design standards | Sanitation technology |
|  | Safe containment of excreta |
|  | Facility accessibility |
|  | Sex segregation |
|  | Privacy |
|  | Menstrual hygiene management facility |
|  | Handwashing facilities |
|  | Pupil to stance ratio |
| Resources management | Trained personnel |
|  | Reliable water supply |
|  | Budget-line implementation |
|  | Material-use tracking system |
| Service delivery | Facility availability |
|  | Facility accessibility |
|  | Facility functionality |
|  | Toilet cleanliness |
|  | Toilet consumables at point of use |
|  | Menstrual hygiene management materials availability |
|  | Safe disposal of solid waste |
|  | Safe excreta disposal |
| Governance | Hygiene promotion activities |
|  | Formal school management structures |
|  | Internal and external stakeholder engagement |
|  | Enforcement |
| Monitoring and evaluation | Use of structured M&E tools |
|  | Multiple stakeholder monitoring |
|  | Making informed decisions from monitoring data |
|  | M&E training of personnel |
|  | Escalation process for unresolved issues |

**Supplementary S2: Questionnaire for Head Teachers / Sanitation Focal Teachers**

**Development of an Operation & Maintenance (O&M) Decision Support Tool for School Sanitation**

Dear Head Teacher or Sanitation Teacher,

Kampala Capital City Authority (KCCA), in collaboration with Makerere University, is developing a Decision Support System (DSS) to strengthen the operation and maintenance (O&M) of school sanitation in Kampala. Your school has been selected to help calibrate the DSS, especially the Likelihood, Severity, and Detection anchors and related status indicators.

**What we ask of you?**

(i) Please complete this questionnaire during the workshop, following the facilitators’ guidance.
(ii) base your answers on what actually happens at your school, using recent records where possible. There are no right or wrong answers.

**Confidentiality & use of data**

Your responses will be used for study and tool-development purposes only. Data will be analyzed in aggregate; no school will be identified in reports without prior permission. All information will be treated as confidential. Before you begin, kindly sign the acceptance/consent form provided. If you have questions, please speak to a workshop facilitator.

Thank you for your contribution to safer, more reliable school sanitation

**A DESIGN WORKSHOP FOR THE DEVELOPMENT OF A SCHOOL SANITATION OPERATION AND MAINTENANCE (O&M) DECISION SUPPORT SYSTEM (DSS)**

**Venue : Mayor’s Polour City Hall**

*Participant Informed Consent Form (ICF)*

*Name : ……………………………………………………………………………………………………...…*

*Position : ……………………………………………………………………………………………………..*

*Profession : ………………………………………………………………………………………..…………*

*Profession experience (Years) :……………………………………………………………………………*

Kampala Capital City Authority (KCCA), in collaboration with Makerere University is developing a Decision Support System (DSS) aimed at improving sanitation operation and maintenance (O&M) in schools across the city. This system will support evidence-based planning, monitoring, and management of sanitation services in schools.

The purpose of this communication is to obtain your consent for participation in a half-day workshop that has brought together selected Head Teachers and Sanitation Teachers from **Central Division** to calibrate the DSS, especially the Likelihood, Severity, and Detection anchors and related status indicators.

*The workshop is taking place on Friday 3rd October 2025 at City Hall, Mayor’s Palour from* *8:00 am to 1:00 pm. Participants will be supported with meals and transport facilitation.*

**What is Involved?**

If you consent to voluntarily participate in this research, your participation will include a questionnaire for you to score based on your expertise and experience on the O & M of a sanitation facility. There will also be a group discussion where your views and those of other experts will be recorded purposely for research purposes. All this will be done during the workshop scheduled timeline

**Participation and Withdrawal**

Your participation in this research is completely voluntary. If you decide to participate you may withdraw at any time without explanation or consequence. You may also skip any or all of the questions in the questionnaire, or any portion of the survey and still collect the compensation as described above.

If you do withdraw from the study after data collection your anonymized data will still be used unless you have requested in writing to have it removed from the database; please email [byansijude@gmail.com](mailto:byansijude@gmail.com) prior to indicate that you are withdrawing from the research and please indicate as appropriate:

1) Your consent for the researchers to use your anonymized collected data in this study
2) Your request to remove your anonymized collected data from the research.

**Anonymity and Confidentiality**

The study team will protect your anonymity by assigning a numeric identification number to the data you provide, which will be stored separately from any of your identifying information, including consent forms. All published data from this study will be in aggregate only (i.e., average performances rather than data from this specific interview). If quotes from your interview are published, they will be published without any identifying information.

All data stored on a computer will be password protected and encrypted. All computers containing data will be password protected. All hard copies of data (i.e., handwritten notes or USB keys) will be kept locked or under direct researcher supervision at all times.

Participant contact information will be kept separately from collected data.

**Contacts**

If you have any questions or concerns about the study, please contact the principal investigator

Eng. Dr. Robinah N. Kulabako

Makerere University

P.O. Box 7062, Kampala

Tel: +256772426621

E-mail: robinah.kulabako@mak.ac.ug or rkulaba@gmail.com

**Consent**

Your signature below indicates that you understand the above conditions of participation in this study, that you have had the opportunity to have your questions answered by the researchers and that you consent to participate in this research project.

| *Name of Participant* |  | *Signature* |  | *Date* |
| --- | --- | --- | --- | --- |

**DEFINITIONS:**

(1) **Sanitation: Provision of facilities and services for the safe disposal and management of human excreta, including containment and treatment to protect health**
(2) **School sanitation (WinS): Clean, private and functional toilets (including provision for MHM) within the school environment to support pupil health, dignity and learning.**
(3) **Operation & Maintenance (O&M): The routine cleaning, resupply, inspection, minor repair, and budgeting required to keep sanitation facilities usable and safe (e.g., rosters, supplies, small fixes, scheduling). Evidence from school trials shows O&M packages (supplies, tools, roles, budgets) improve toilet usability (accessible, functional, private).**
(4) **Joint Monitory Program (JMP) service levels (Sanitation, in schools): Global monitoring classes used by WHO/UNICEF:**

| **#** | **Service level** | **Details** |
| --- | --- | --- |
| **1** | **Basic** | **Improved sanitation facilities that are available, accessible, functional , private and single-sex for pupils** |
| **2** | **Limited** | **Improved sanitation facilities that miss one or more characteristic for basic sanitation service** |
| **3** | **No Service** | **Unimproved sanitation facilities in use or no sanitation facilities for pupils.** |

(5) **Menstrual hygiene management (MHM) facility:** A private, functional space where girls can manage menstruation with materials, water, soap, and safe disposal.
(6) **Pupil-to-toilet stance ratio (PSR):** The number of pupils per usable stance; In Uganda PRS should not exceed 40:1
(7) **Domains (in this tool):** Thematic groupings that organize indicators. For this study we have six domains namely: Service Planning, Facility Design Standards, Resource Management, Service Delivery, Governance, Monitoring & Evaluation
(8) **Indicator**: A quantitative or qualitative measure used to track a construct (status, process, or outcome) and inform decisions.
(9) **Indicator status**: Indicator status refers to the level of performance of a sanitation indicator relative to expected service standards, measured on a five-point scale ranging from poor (non-functional or absent) to optimal (fully implemented and continuously improved). It reflects the extent to which the indicator is available, functional, consistently implemented, and supported by monitoring and management systems.

| **Score (% of expected service)** | **Score** | **Definition** |
| --- | --- | --- |
| **1 (0–20%)** | **Poor / Not meeting service standard** | The indicator is absent, non-operational, or in very poor condition; no evidence of implementation, control, or record (e.g., toilets in very dirty condition or absence of M&E training). |
| **2 (21–40%)** | **Limited / Partially meeting service standard** | The indicator exists but is inconsistent, irregular, or inadequately implemented; performance is below acceptable levels with minimal control or documentation (e.g., intermittent water supply or irregular cleaning practices). |
| **3 (41–60%)** | **Basic / Meeting minimum service standard** | The indicator is in place and meets the minimum expected standard, though gaps remain in consistency, coverage, or quality (e.g., pupil-to-stance ratio meeting the minimum standard but with congestion at peak times). |
| **4 (61–85%)** | **Advanced / Well-performing and reliable** | The indicator is consistently implemented, adequately resourced, and routinely monitored; performance is stable and reliable (e.g., regular cleaning schedules with supervision). |
| **5 (86–100%)** | **Optimal / Exemplary and improving** | The indicator reflects best practice, with full institutionalization, documentation, routine monitoring, and continuous improvement mechanisms (e.g., use of structured M&E tools with feedback loops and continuous improvement). |

(10) **Likelihood (risk):** A quantitative or qualitative measure used to track a construct (status, process, or outcome) and inform decisions.

| **Score** | **Likelihood level** | **Implication** | **Description (Lane & Hrudey 2023)** |
| --- | --- | --- | --- |
| **1** | **Very unlikely** | **0 - 20% probable** | **Once every 5 years** |
| **2** | **Unlikely** | **21 - 40% probable** | **Once every year** |
| **3** | **Possible** | **41 -60% probable** | **Once every month** |
| **4** | **Likely** | **61 – 80% probable** | **Once every week** |
| **5** | **Very likely** | **86 - 100% probable** | **Once everyday** |

(11) **Severity (risk): Magnitude of impact on sanitation service and pupils if the failure occurs**

| **Level** | **Descriptor** | **Definition** | **Operational Impact** | **Example in School Sanitation Context** |
| --- | --- | --- | --- | --- |
| **1** | **Insignificant** | Event has no noticeable effect on the sanitation system or service continuity. | Minor inconvenience only. | Minor dripping tap; faint odor near a functional toilet. |
| **2** | **Minor** | Slight degradation of facility performance; easily corrected through normal maintenance. | Minimal disruption, localized issue. | Blocked handwashing tap; one cubicle temporarily out of use. |
| **4** | **Moderate** | Noticeable reduction in functionality or hygiene standards for a group of users. | Localized disruption; may affect access or usability. | Toilets for one gender locked; water unavailable in one block. |
| **8** | **Major** | Significant breakdown affecting usability, privacy, or hygiene for many users. | Major disruption to school routines or gender access. | Wastewater overflow in one block; absence of privacy doors; multiple toilets non-functional. |
| **16** | **Catastrophic** | Complete system failure or serious event causing widespread risk or closure. | Entire sanitation block or school operations disrupted; requires emergency action. | Sewage backflow, collapsed pit slab, or total water outage for the whole school. |

(12) **Detection (risk): How early and reliably the school can notice and act to prevent or limit the failure; lower scores = easier detection.**

| **Score** | **Severity level** | **Implication** |
| --- | --- | --- |
| **1** | **Very easy** | **Detected during pre-term review meeting that always happens 14 days before opening of term** |
| **2** | **Easy** | **Detected during the routine check done 8–13 days before term begins** |
| **3** | **Moderate** | **Detected during a formal check that is done 3–7 days before term begins** |
| **4** | **Difficult** | **Detected 2 - 3 days before term begins using none routine review methods** |
| **5** | **Very difficult** | **Problems noticed after classes begin or via complaints** |
| **6** | **Almost impossible** | **Discovered only after major disruption or external inspection for there are no records or review of records.** |

**1.0	SCHOOL DETAILS**

1.1 What is the name of the school?

……………………………………………………………………………………………………

1.2 State the address of your schools

……………………………………………………………………………………………………

1.3 State the telephone number of your Head Teacher:

……………………………………………………………………………………………………

1.4 What is the Education level of this school?

❑ Primary (1)	❑ Secondary (2)

1.5 Sate the type of school by funding:

❑ Government	❑ Private

1.6 In which Division is your school found:

❑	Central Division (1)	❑ Kawempe Division (2)	❑ Makindye Division (3)

❑ Nakawa Division (4)	❑ Rubaga Division (5)

1.7 State the school type by mode of attendance:

❑ Day primary school ❑ Boarding Primary school, ❑ Day and boarding primary school

❑ Day secondary school ❑ Boarding secondary school, ❑ Day and boarding secondary school

1.8 State the school type by gender:

❑ Girls’ school only	❑ Boy’s school only ❑ Mixed school

1.9 What is the school pupil population? ……………………………………………………..
1.10 Number of Students/Pupils: Male: ………… Female: …………… Total: ………………
1.11 What is the total number of toilet stances for pupils in your school? ……………….……
1.12 How many toilet stances are used by Boys only: ………….. Girls only: ……………., shared by boys and girls: …………….
1.13 Determine the Pupil to stance ratio = Total number of pupils/Total number of toilet stances

……………………………………………………………………………………………………

1.14 Number of teaching staff: Male: ………… Female: ……………Total: …………….……
1.15 How many toilet stances are used by: male teachers only: ………… female teachers only: ……………., shared by male and female teachers: ………………………………...……...

**2.0	SERVICE PLANNING DOMAIN ASSESSMENT**

2.1 (a) Does the school have a documented Operation and maintenance (O&M) plan for sanitation facilities?

❑ School has no O&M plan in place (Status score: ----).

❑ O&M plan exists but it is not updated (Status score: ----).

❑ O&M plan exists and in use (Status score: ----).

| **Category** | **Question** | **Score** |
| --- | --- | --- |
| (b) Likelihood | In the next school term, what is the likelihood that this school will start the term without an updated O&M plan? |  |
| (c) Severity | If the school starts a term without an O&M plan, how severe is the disruption to the service? |  |
| (d) Detection | How easy would it be to notice before classes begin that the school will start the term without an updated O&M plan? |  |

2.2 (a) Are O&M roles and responsibilities clearly assigned, documented, and functional?

❑ No O&M roles assigned or they are not documented (Status score: ----).

❑ Roles are documented but not assigned or followed (Status score: ----).

❑ Roles are clear, documented, and actively followed (Status score: ----).

| **Category** | **Question** | **Score** |
| --- | --- | --- |
| (b) Likelihood | In the next school term, what is the likelihood that the school will operate without clearly assigned, documented, and functioning O&M roles (e.g., cleaning lead, maintenance focal person, supplies/stock checks)? |  |
| (c) Severity | If O&M roles are unclear or not functioning, how bad would the impact be on pupils’ sanitation services (access, privacy, cleanliness, water/soap)? |  |
| (d) Detection | How easy would it be to notice before classes begin in the new term that O&M roles are unclear or not functioning? |  |

2.3 (a) Does the school have a dedicated and functional budget-line for sanitation O&M?

❑ No dedicated budget-line exists (Status score: ----).

❑ Budget-line exists but funds are irregular or not disbursed (Status score: ----).

❑ Budget-line exists and is regularly funded and used (Status score: ----).

| **Category** | **Question** | **Score** |
| --- | --- | --- |
| (b) Likelihood | In the next school term, what is the likelihood that the school will start the term without a dedicated sanitation O&M budget-line properly set up and usable? |  |
| (c) Severity | If the school starts a term without a dedicated O&M budget-line, how bad would the impact be on pupils’ sanitation services (access, privacy, cleanliness, water/soap)? |  |
| (d) Detection | How easy would it be to notice before classes begin that there is no dedicated O&M budget-line set up, and to act in time to prevent service problems? |  |

2.4 (a) Does the school implement a preventive maintenance schedule for sanitation facilities?

❑ No preventive maintenance schedule exists (Status score: ----).

❑ Schedule exists but not consistently implemented (Status score: ----).

❑ Schedule exists and is implemented regularly (Status score: ----).

| **Category** | **Question** | **Score** |
| --- | --- | --- |
| (b) Likelihood | In the next school term, what is the likelihood that the school will miss or not implement the preventive maintenance schedule for sanitation facilities? |  |
| (c) Severity | If the preventive maintenance schedule is not implemented on time, how bad would the impact be on pupils’ sanitation services (access, privacy, cleanliness, water/soap)? |  |
| (d) Detection | How easy would it be to notice before problems arise that preventive maintenance will not be done on time, and to act early to prevent service issues? |  |

**3.0	FACILITY DESIGN STANDARDS DOMAIN ASSESSMENT**

3.1 (a) Do all sanitation facilities in the school meet JMP standards for improved technology?

❑ No sanitation facilities in the school use improved technologies. All are unimproved or unsafe. (Status score: ----).

❑ Some sanitation facilities in the school use improved technologies, while others are unimproved. (Status score: ----).

❑ All sanitation facilities in the school use improved technologies (e.g., flush/pour-flush, VIP, pit latrine with slab, composting toilet). (Status score: ----).

| **Category** | **Question** | **Score** |
| --- | --- | --- |
| (b) Likelihood | In the next school term, what is the likelihood that the school will have one or more sanitation facilities that are not improved technology? |  |
| (c) Severity | If one or more facilities are not improved technology, how bad would the impact be on pupils’ sanitation services (access, privacy, cleanliness, water/soap)? |  |
| (d) Detection | How easy would it be to notice before classes begin that at least one facility is not improved technology, and to act early (e.g., schedule upgrade/temporary controls/alternative arrangements) to avoid service problems? |  |

3.2 (a) Do all school sanitation systems safely contain excreta without visible leakage or environmental contamination?

❑ Multiple toilets have leaking pits/tanks or visible signs of seepage/overflow into the environment or pupils are practicing open defecation while at school (Status score: ----).

❑ Some toilets are affected by leakage or poor containment, but majority function well. (Status score: ----).

❑ All toilets are free from leakage or seepage and safely contain excreta (Status score: ----).

| **Category** | **Question** | **Score** |
| --- | --- | --- |
| (b) Likelihood | In the next school term, what is the likelihood that one or more school toilets will have leakage, seepage, or overflow of excreta to the environment (or that pupils will defecate in the open on school grounds)? |  |
| (c) Severity | If leakage/seepage/overflow occurs, how bad would the impact be on pupils’ sanitation services (access, privacy, cleanliness, water/soap)? |  |
| (d) Detection | How easy would it be to notice in time that a leakage/overflow is about to happen (e.g., rising pit/tank level, soak-away failure), and act early (desludge/repair/cordon) to prevent service problems? |  |

3.3 (a) Are sanitation facilities inclusive, accessible, and functional for pupils with disabilities?

❑ No disability-friendly facilities (Status score: ----).

❑ Facilities exist but not functional or inadequate (Status score: ----).

❑ Accessible and functional facilities available (Status score: ----).

| **Category** | **Question** | **Score** |
| --- | --- | --- |
| (b) Likelihood | In the next school term, what is the likelihood that the school will start the term without an accessible and functional disability-friendly toilet for pupils with disabilities? |  |
| (c) Severity | If there is no accessible and functional disability-friendly toilet, how bad would the impact be on pupils’ sanitation services? |  |
| (d) Detection | How easy would it be to notice before classes begin that the accessible toilet or its approach is not usable (e.g., blocked path, broken ramp/grab bars, unreachable fixtures), and to fix it in time? |  |

3.4 (a) Are toilets sex-segregated and used separately by boys and girls?

❑ No sex segregation (Status score: ----).

❑ Partial segregation or shared use (Status score: ----).

❑ Full segregation and designated use (Status score: ----).

| **Category** | **Question** | **Score** |
| --- | --- | --- |
| (b) Likelihood | In the next school term, what is the likelihood that toilets will not be fully sex-segregated and used separately (e.g., missing/unclear signage, shared use, cross-use at breaks)? |  |
| (c) Severity | If toilets are not fully sex-segregated/used separately, how bad would the impact be on pupils’ sanitation services (privacy, access, cleanliness, water/soap)? |  |
| (d) Detection | How easy would it be to notice before or in the first week that sex-segregation arrangements are not in place or not being followed, and to fix them in time? |  |

3.5 (a) Do all toilet stances have functional doors and locks that guarantee privacy?

❑ (i) Most toilet stances lack doors/locks (Status score: ----).

❑ (ii) More than half of toilets have doors/locks but some lack privacy (Status score: ----).

❑ (iii) All toilets have functional doors/locks (Status score: ----).

| **Category** | **Question** | **Score** |
| --- | --- | --- |
| (b) Likelihood | In the next school term, what is the likelihood that one or more toilet stances will not provide privacy (no door, no working lock, or large gaps)? |  |
| (c) Severity | If some stances do not provide privacy, how bad would the impact be on pupils’ sanitation services? |  |
| (d) Detection | How easy would it be to notice before or in the first week that doors/locks/privacy are not in place or not working, and to fix them in time? |  |

3.6 (a) Does the school provide private, functional, and adequately equipped facilities for menstrual hygiene management (MHM)?

❑ No MHM facilities exist or they exist but are non-functional and non-private (Status score: ----).

❑ Some MHM facilities exist but lack either privacy, functionality, or essential supplies (Status score: ----).

❑ Fully equipped, private, and functional MHM facilities are available and accessible to girls (Status score: ----).

| **Category** | **Question** | **Score** |
| --- | --- | --- |
| (b) Likelihood | In the next school term, what is the likelihood that the school will start the term without a private, functional, and adequately supplied MHM facility accessible to girls? |  |
| (c) Severity | If there is no private, functional, and adequately supplied MHM facility, how bad would the impact be on pupils’ sanitation services? |  |
| (d) Detection | How easy would it be to notice before classes begin that the MHM facility won’t be private/functional/supplied, and to act in time (stocking, fixing locks/water, arranging disposal)? |  |

3.7 (a) Are functional handwashing facilities with water and soap available near all toilets for all users?

❑ No handwashing stations near toilets (Status score: ----).

❑ Stations exist but lack water or soap (Status score: ----).

❑ Fully functional handwashing stations available and accessible (Status score: ----).

| **Category** | **Question** | **Score** |
| --- | --- | --- |
| (b) Likelihood | In the next school term, what is the likelihood that the school will start the term without functional handwashing (water and soap) near all toilets for all users? |  |
| (c) Severity | If functional handwashing with water and soap is not available near some toilets, how bad would the impact be on pupils’ sanitation services? |  |
| (d) Detection | How easy would it be to notice before classes begin that HW points near toilets won’t be functional and stocked (water and soap), and to act in time (repairs, refills, repositioning)? |  |

3.8 (a) Does the school meet the recommended pupil-to-toilet stance ratio standard (≤40:1)?

❑ Ratios far exceed recommended standards (above 50:1) (Status score: ----).

❑ Close to recommended but slightly high: (PSR not exceeding 50:1) (Status score: ----).

❑ Meets recommended ratios (Less than or equal to 40:1) (Status score: ----).

| **Category** | **Question** | **Score** |
| --- | --- | --- |
| (b) Likelihood | In the next school term, what is the likelihood that the school will start the term with a PSR above 40:1 for any pupil group? |  |
| (c) Severity | If the school starts the term with PSR >40:1 (for any group), how bad would the impact be on pupils’ sanitation services? |  |
| (d) Detection | How easy would it be to notice before classes begin that PSR will be >40:1 for any group, and to act in time (open extra stances, stagger breaks, fast repairs)? |  |

**4.0	RESOURCES MANAGEMENT DOMAIN ASSESSMENT**

4.1 (a) Has the school designated and trained staff to manage sanitation O&M on a continuous basis?

❑ No trained personnel and no training conducted (Status score: ----).

❑ Staff are assigned but not trained or trained once (Status score: ----).

❑ Designated trained staff with periodic training (Status score: ----).

| **Category** | **Question** | **Score** |
| --- | --- | --- |
| (b) Likelihood | In the next school term, what is the likelihood that the school will start the term without designated, trained staff actively managing sanitation O&M? |  |
| (c) Severity | If there are no designated, trained O&M staff, how bad would the impact be on pupils’ sanitation services (access, privacy, cleanliness, water/soap)? |  |
| (d) Detection | How easy would it be to notice before classes begin that no designated, trained O&M staff will be in place, and to act in time (assign, train, arrange cover)? |  |

4.2 (a) Is safe and reliable water consistently available in the school for sanitation and hygiene purposes?

❑ No water sources exist on school compound or supply is completely unavailable (Status score: ----).

❑ Water is intermittently available or supply is unreliable during parts of the day/week. (Status score: ----).

❑ Water is consistently available at the school compound for all sanitation needs throughout the school day. (Status score: ----).

| **Category** | **Question** | **Score** |
| --- | --- | --- |
| (b) Likelihood | In the next school term, what is the likelihood that the school will start the term without reliable water available all day for sanitation and hygiene (toilets, cleaning, handwashing)? |  |
| (c) Severity | If reliable water is not available all day, how bad would the impact be on pupils’ sanitation services (access, cleanliness, handwashing)? |  |
| (d) Detection | How easy would it be to notice before classes begin that water won’t be reliably available all day, and to act in time (refill/repair/vendor delivery)? |  |

4.3 (a) Is the sanitation O&M budget regularly allocated, disbursed, and utilized and tracked?

❑ No budget allocated (Status score: ----).

❑ Budget exists but under used (Status score: ----).

❑ Budget used and tracked (Status score: ----).

| **Category** | **Question** | **Score** |
| --- | --- | --- |
| (b) Likelihood | In the next school term, what is the likelihood that the school will start the term without an allocated, disbursed, and tracked sanitation O and M budget? |  |
| (c) Severity | If the sanitation O and M budget is not allocated, disbursed, or tracked, how bad would the impact be on pupils’ sanitation services such as access, privacy, cleanliness, water and soap, and minor repairs? |  |
| (d) Detection | How easy would it be to notice before classes begin that the sanitation O and M budget has not been allocated, disbursed, or recorded in the tracking system, and to act in time to approve, release, or enter the funds? |  |

4.4 (a) Does the school have a structured system to manage sanitation material supplies (procurement, storage, distribution, and replenishment)?

❑ No evidence of a supply management system; materials are sourced ad hoc. (Status score: ----)

❑ A partial or informal system exists, but it is inconsistently applied. (Status score: ----).

❑ A clear, structured system exists and is actively used to manage sanitation supplies (Status score: ----).

| **Category** | **Question** | **Score** |
| --- | --- | --- |
| (b) Likelihood | In the next school term, what is the likelihood that the school will start the term without a structured system in place to manage sanitation material supplies (procurement, storage, distribution, and replenishment)? |  |
| (c) Severity | If there is no structured system for sanitation material supplies, how bad would the impact be on pupils’ sanitation services such as toilet use, cleanliness, handwashing with soap, and menstrual hygiene management? |  |
| (d) Detection | How easy would it be to notice before classes begin that the sanitation material supply system is missing or not functional, and to act in time (e.g., purchase, restock, or arrange alternative supply)? |  |

**5.0	SANITATION SERVICE DELIVERY DOMAIN ASSESSMENT**

5.1 (a) Are toilets present on the school premises and available for use by all pupils throughout the school day without restriction or reservation?

❑ Toilets are not available or are locked/reserved, making them unusable by most pupils (Status score: ----).

❑ Toilets are available but access is restricted to certain groups or times. (Status score: ----).

❑ Toilets are present and open to all pupils during school hours without restriction (Status score: ----).

| **Category** | **Question** | **Score** |
| --- | --- | --- |
| (b) Likelihood | In the next school term, what is the likelihood that the school will start the term with toilets not freely available to all pupils (e.g., locked, ‘staff only’, restricted hours, key not accessible)? |  |
| (c) Severity | If toilets are not freely available to all pupils, how bad would the impact be on pupils’ sanitation services? |  |
| (d) Detection | How easy would it be to notice before term begins that toilets won’t be freely available to all pupils (e.g., locked, key controls, staff-only rules), and to act in time to prevent access problems? |  |

5.2 (a) Are toilets open and accessible to all pupils during school hours (not locked or restricted)?

❑ Toilets often locked or difficult to access (Status score: ----).

❑ Access inconsistent or partial (Status score: ----).

❑ Fully accessible during all hours (Status score: ----).

| **Category** | **Question** | **Score** |
| --- | --- | --- |
| (b) Likelihood | In the next school term, what is the likelihood that toilets will not be open and accessible to all pupils during school hours (locked, key unavailable, restricted times/groups)? |  |
| (c) Severity | If toilets are not open/accessible to all pupils during school hours, how bad would the impact be on pupils’ sanitation services? |  |
| (d) Detection | How easy would it be to notice before classes begin that toilets won’t be open/accessible (locked, key controls, restrictions), and to act in time to prevent access problems? |  |

5.3 (a) What proportion of toilets are functional, safe, and usable by pupils at the time of assessment?

❑ Less than 50% functional (Status score: ----).

❑ 50–74% functional (Status score: ----).

❑ 75% or more functional (Status score: ----).

| **Category** | **Question** | **Score** |
| --- | --- | --- |
| (b) Likelihood | In the next school term, what is the likelihood that the school will start the term with <75% of stances functional, safe, and usable? |  |
| (c) Severity | If less than 75% of stances are functional/usable, how bad would the impact be on pupils’ sanitation services? |  |
| (d) Detection | How easy would it be to notice before term begin that less than 75% of stances will be functional/usable, and to act in time (repairs, open backups, water provisioning)? |  |

5.4 (a) Are toilets consistently kept visually clean, odor-free, and hygienic at the time of inspection?

❑ Toilets are consistently dirty or smelly (Status score: ----).

❑ Occasional unclean state (Status score: ----).

❑ Toilets always clean and odor-free (Status score: ----).

| **Category** | **Question** | **Score** |
| --- | --- | --- |
| (b) Likelihood | In the next school term, what is the likelihood that the school will start the term without a functioning sanitation supplies management system (so that key items are not planned/ordered/stored), leading to stock-outs? |  |
| (c) Severity | If toilets are not kept clean/odor-free, how bad would the impact be on pupils’ sanitation services? |  |
| (d) Detection | How easy would it be to notice before classes begin that cleanliness/odor control won’t be maintained (e.g., no roster, no supplies), and to act in time (assign cleaners, stock supplies, schedule rounds)? |  |

5.5 (a) Are essential consumables (soap, toilet paper, pads, disinfectants) consistently available at the point of use?

❑ No consumables are available at sanitation points. (Status score: ----).

❑ Some consumables are available, but not consistently or not for all users. (Status score: ----).

❑ All required consumables are regularly available and accessible to all users (Status score: ----).

| **Category** | **Question** | **Score** |
| --- | --- | --- |
| (b) Likelihood | In the next school term, what is the likelihood that the school will start the term without essential consumables consistently available at the point of use (soap at HW points, tissue per policy, pads in MHM room, disinfectants for cleaning)? |  |
| (c) Severity | If essential consumables are not consistently available at point of use, how bad would the impact be on pupils’ sanitation services? |  |
| (d) Detection | How easy would it be to notice before classes begin that essential consumables won’t be in place at point of use, and to act in time (procure, restock, redistribute)? |  |

5.6 (a) Does the school provide girls with regular access to safe and adequate menstrual hygiene materials?

❑ No menstrual materials are provided (pads/cloths), or stock-outs are the norm (Status score: ----).

❑ Materials exist but supply is irregular, inadequate, or not accessible to all girls (Status score: ----).

❑ Materials are consistently available, sufficient, and accessible to all girls who need them (Status score: ----).

| **Category** | **Question** | **Score** |
| --- | --- | --- |
| (b) Likelihood | In the next school term, what is the likelihood that girls will not have regular access to safe and adequate menstrual materials (due to stock-outs, insufficient quantities, or access barriers)? |  |
| (c) Severity | If menstrual materials are not regularly available/accessible, how bad would the impact be on pupils’ sanitation services and participation (privacy, dignity, attendance)? |  |
| (d) Detection | How easy would it be to notice before classes begin that menstrual materials won’t be available/sufficient/accessible, and to act in time (procure, restock, organize distribution)? |  |

5.7 (a) Does the school safely manage solid waste through segregation, protected storage, and safe disposal without open dumping or burning?

❑ School dumps or burns solid waste in the open; no segregation or proper bins used (Status score: ----).

❑ School collects waste in bins but lacks segregation or safe final disposal mechanism (Status score: ----).

❑ School segregates, collects, and safely disposes of waste or has evidence of plans to implement such a system (Status score: ----).

| **Category** | **Question** | **Score** |
| --- | --- | --- |
| (b) Likelihood | In the next school term, what is the likelihood that the school will not manage solid waste safely (no segregation/protected storage/safe disposal), leading to open dumping or burning? |  |
| (c) Severity | If solid waste is not managed safely (no segregation/protected storage/safe disposal), how bad would the impact be on pupils’ sanitation services and school environment? |  |
| (d) Detection | How easy would it be to notice before classes begin that safe solid-waste management won’t be in place (no segregation/storage/disposal arrangements), and to act in time (bins, storage, collection/contract)? |  |

5.8 (a) Does the school safely manage the disposal of excreta or feacal sludge using acceptable desludging methods and without unsafe practices? The unsafe feacal sludge disposal methods include:

(i) dumping feacal sludge into the environment, ditches, or storm water drains;

(ii) open-air drying of feacal sludge without containment or treatment;

(iii) unprotected manual emptying without personal protective equipment (PPE); or (iv) burial or spreading of feacal sludge in open fields.

❑ School practices or shows evidence of any unsafe feacal sludge disposal methods (Status score: ----).

❑ School has no current unsafe practices, but lacks documented safe desludging methods or plans (Status score: ----).

❑ School uses or has documented plans for safe desludging, and avoids all forms of unsafe disposal (Status score: ----).

| **Category** | **Question** | **Score** |
| --- | --- | --- |
| (b) Likelihood | In the next school term, what is the likelihood that the school will use an unsafe feacal sludge disposal practice or be forced into one (e.g., dumping, unprotected manual emptying, uncontrolled drying/burial)? |  |
| (c) Severity | If an unsafe FS disposal occurs, how bad would the impact be on pupils’ sanitation services and school environment? |  |
| (d) Detection | How easy would it be to notice before problems arise that safe desludging won’t be in place (no contract/receipt, capacity risk, no PPE/SOP), and to act in time (book service, arrange safe manual emptying, containment)? |  |

**5.0	GOVERNANCE DOMAIN ASSESSMENT**

6.1 (a) Does the school implement regular and structured hygiene promotion activities that increase sanitation-related knowledge and awareness among pupils and staff?

❑ No evidence or irregular promotion of sanitation and hygiene knowledge within the school (No hygiene education sessions conducted, no sanitation-related posters or teaching aids visible., No sanitation or hygiene topics at the assemblies) (Status score: ----).

❑ Some sanitation and hygiene promotion activities exist but are irregular or limited (Occasional hygiene talks or events, Some posters or instructional materials in classrooms or toilets, One-off lessons or initiatives without routine integration) (Status score: ----).

❑ Regular and structured sanitation and hygiene awareness activities are integrated into school life (Hygiene education integrated in the curriculum or routine health education, posters, reminders, or learning materials consistently displayed in key areas, Pupils receive regular sanitation messaging through clubs, assemblies, or lessons.) (Status score: ----).

| **Category** | **Question** | **Score** |
| --- | --- | --- |
| (b) Likelihood | In the next school term, what is the likelihood that the school will not implement regular, structured hygiene-promotion activities (curriculum/assemblies/clubs with visible materials)? |  |
| (c) Severity | If regular, structured hygiene promotion is not implemented, how bad would the impact be on pupils’ sanitation services and behaviors (handwashing with soap, proper toilet use/cleanliness, MHM practices)? |  |
| (d) Detection | How easy would it be to notice before classes begin that hygiene-promotion activities won’t be in place (no schedule/materials/club plan), and to act in time (schedule sessions, brief staff, refresh posters)? |  |

6.2 Does the school have the following key institutional structures in a functioning state to support sanitation operation and maintenance? (i) School Management Committee (ii) Sanitation committee (iii) Sanitation teacher (focal person)

❑ None or only one of the four key structures is in place or functional (Status score: ----).

❑ Two or three of the key structures are in place and at least partially functional (Status score: ----).

❑ All four key structures are in place and actively involved in coordinating, implementing, and overseeing sanitation O&M activities (Status score: ----).

| **Category** | **Question** | **Score** |
| --- | --- | --- |
| (b) Likelihood | In the next school term, what is the likelihood that the school will have fewer than all four key structures in place and functioning (i.e., one or more missing/inactive)? |  |
| (c) Severity | If one or more of the key structures are not in place or not functioning, how bad would the impact be on pupils’ sanitation services (access, privacy, cleanliness, water/soap, response to issues)? |  |
| (d) Detection | How easy would it be to notice before classes begin that one or more of the four structures won’t be in place or functioning (no TORs, no calendar, no members), and to act in time (appoint, brief, schedule)? |  |

6.3 (a) Does the school regularly involve internal (teachers, pupils, clubs) and external stakeholders (parents, community leaders, NGOs, local government) in the planning, implementation, and evaluation of sanitation services?

❑ No or minimal stakeholder involvement beyond school administration (teachers, pupils and clubs) (Status score: ----).

❑ Some external stakeholders are involved but irregularly or in a limited manner (e.g. only parents, one NGO) (Status score: ----).

❑ Multiple stakeholder groups are regularly and actively engaged, with records of meetings and actions (Status score: ----).

| **Category** | **Question** | **Score** |
| --- | --- | --- |
| (b) Likelihood | In the next school term, what is the likelihood that the school will not regularly involve multiple internal and external stakeholders in sanitation planning/implementation/evaluation? |  |
| (c) Severity | If multiple stakeholder groups are not regularly engaged, how bad would the impact be on pupils’ sanitation services (supplies, repairs, cleanliness, water/soap, behavior support)? |  |
| (d) Detection | How easy would it be to notice before classes begin that multi-stakeholder involvement won’t be in place (no calendar/minutes/contacts), and to act in time (schedule meetings, invite partners, assign actions)? |  |

6.4 (a) Does the school have clear sanitation-related rules and ensure their consistent enforcement among pupils and staff?

❑ No clear sanitation related rules and no enforcement among pupils? (Status score: ----).

❑ Rules exists but no enforcement (Status score: ----).

❑ Rules exists and are enforced (Status score: ----).

| **Category** | **Question** | **Score** |
| --- | --- | --- |
| (b) Likelihood | In the next school term, what is the likelihood that the school will not consistently enforce sanitation rules using the agreed tools (rosters, checklists, logs)? |  |
| (c) Severity | If sanitation rules are not consistently enforced with tools, how bad would the impact be on pupils’ sanitation services and behaviors (proper toilet use, privacy, cleanliness, handwashing)? |  |
| (d) Detection | How easy would it be to notice before classes begin that the rules & enforcement system won’t be in place (no posters/rosters/checklists/logs), and to act in time (post rules, assign duty, start logs)? |  |

**5.0	MONITORING AND EVALUATION DOMAIN ASSESSMENT**

7.1 (a) Are sanitation facilities monitored on a routine basis using standardized checklists or structured M&E tools, with records kept?

❑ No monitoring tools are used (only informal observation) (Status score: ----).

❑ Tools exist but are inconsistently or irregularly applied (Status score: ----).

❑ Tools are regularly and systematically applied, with records kept (Status score: ----).

| **Category** | **Question** | **Score** |
| --- | --- | --- |
| (b) Likelihood | In the next school term, what is the likelihood that the school will not conduct routine monitoring of sanitation using standardized checklists with records (and an action tracker)? |  |
| (c) Severity | If routine monitoring with standardized tools does not happen, how bad would the impact be on pupils’ sanitation services (access, privacy, cleanliness, water/soap, timely repairs)? |  |
| (d) Detection | How easy would it be to notice before classes begin that the monitoring system (checklists, schedule, records, action tracker) won’t be in place, and to act in time (assign staff, print tools, set schedule)? |  |

7.2 (a) Do monitoring visits consistently involve multiple stakeholders, including pupils, School Management Committees (SMCs), teachers, and external community representatives?

❑ No stakeholder participation (Status score: ----).

❑ Limited or one-off participation (Status score: ----).

❑ Regular involvement of diverse stakeholders (Status score: ----).

| **Category** | **Question** | **Score** |
| --- | --- | --- |
| (b) Likelihood | In the next school term, what is the likelihood that monitoring visits will not include multiple stakeholder groups (pupils, SMC, teachers, external representatives)? |  |
| (c) Severity | If monitoring does not include multiple stakeholder groups, how bad would the impact be on pupils’ sanitation services (issue detection, accountability, speed of fixes, behavior support)? |  |
| (d) Detection | How easy would it be to notice before classes begin that multi-stakeholder participation in monitoring won’t be in place (no invites/attendance plans), and to act in time (schedule, invite, brief roles)? |  |

7.3 (a) Are records from monitoring visits documented, reviewed, and used systematically to guide improvements in sanitation O&M?

❑ No monitoring records or use of data (Status score: ----).

❑ Records kept but rarely used (Status score: ----).

❑ Records actively inform O&M improvements (Status score: ----).

| **Category** | **Question** | **Score** |
| --- | --- | --- |
| (b) Likelihood | In the next school term, what is the likelihood that monitoring records will not be documented, reviewed, and used systematically to guide O&M improvements? |  |
| (c) Severity | If monitoring records are not documented/reviewed/used, how bad would the impact be on pupils’ sanitation services (repairs, cleanliness, water/soap, privacy)? |  |
| (d) Detection | How easy would it be to notice before classes begin that records won’t be documented/reviewed/used (no schedule, no tracker, no recent records), and to act in time (set schedule, assign roles, start tracker)? |  |

7.4 (a) Have sanitation focal teachers and relevant staff received recent, structured training in sanitation monitoring and inspection?

❑ No training provided (Status score: ----).

❑ Informal or outdated training (Status score: ----).

❑ Recent and relevant training completed (Status score: ----).

| **Category** | **Question** | **Score** |
| --- | --- | --- |
| (b) Likelihood | In the next school term, what is the likelihood that focal teachers and relevant staff will not have recent, structured training in monitoring/inspection (so checks are weak or inconsistent)? |  |
| (c) Severity | If staff do not have recent, structured training in monitoring/inspection, how bad would the impact be on pupils’ sanitation services (timely detection, cleanliness, water/soap, repairs)? |  |
| (d) Detection | How easy would it be to notice before classes begin that training won’t be in place/adequate (no calendar, no recent session, no materials/assessment), and to act in time (schedule refresher/induction)? |  |

7.5 (a) Does the school have a documented and functional process for escalating unresolved sanitation issues to higher authorities within or outside the school?

❑ No escalation process (Status score: ----).

❑ Escalation exists but not documented or followed (Status score: ----).

❑ Escalation process is documented and active (Status score: ----).

| **Category** | **Question** | **Score** |
| --- | --- | --- |
| (b) Likelihood | In the next school term, what is the likelihood that unresolved sanitation issues will not be escalated effectively (no/unclear process, outdated contacts, no follow-through)? |  |
| (c) Severity | If escalation does not work (delays/no action), how bad would the impact be on pupils’ sanitation services (access, cleanliness, water/soap, safety)? |  |
| (d) Detection | How easy would it be to notice before term begins that the escalation system won’t function (SOP missing, contacts outdated, no SLA/log), and to act in time (update contacts, assign lead, agree response times)? |  |

**Supplementary material S3: Semi-quantitative risk assessment matrix**

**Table 6. Semi-quantitative risk assessment matrix** (Adopted from WHO, (2022))

|  |  | **Severity (S)** | | | | | | | |
| --- | --- | --- | --- | --- | --- | --- | --- | --- | --- |
|  |  | **Insignificant (1)** | **Minor (2)** | | **Moderate (4)** | | **Major (8)** | | **Catastrophic (16)** |
| **Likelihood (L)** | **Very unlikely (1)** | 1 | 2 | | 4 | | 8 | | 16 |
|  | **Unlikely (2)** | 2 | 4 | | 8 | | 16 | | 32 |
|  | **Possible (3)** | 3 | 6 | | 12 | | 24 | | 48 |
|  | **Likely (4)** | 4 | 8 | | 16 | | 32 | | 64 |
|  | **Almost certain (5)** | 5 | 10 | | 20 | | 40 | | 80 |
| **Risk score= L x S** | | <6 | | 6 - 12 | | 13 - 32 | | >32 | |
| **Risk level** | | Low risk | | Medium risk | | High risk | | Very high | |

**Supplementary material S4: Status level profiles of operation and maintenance indicators across schools**

***Figure 3. Status level profiles of operation and maintenance indicators across schools, ordered by the combined proportion of poor and limited responses***.

**Supplementary material S4: Semi-quantitative risk assessment matrix**

**Table 3. Semi-quantitative risk assessment matrix** (Adopted from WHO, (2022))

|  |  | **Severity (S)** | | | | | | | |
| --- | --- | --- | --- | --- | --- | --- | --- | --- | --- |
|  |  | **Insignificant (1)** | **Minor (2)** | | **Moderate (4)** | | **Major (8)** | | **Catastrophic (16)** |
| **Likelihood (L)** | **Very unlikely (1)** | 1 | 2 | | 4 | | 8 | | 16 |
|  | **Unlikely (2)** | 2 | 4 | | 8 | | 16 | | 32 |
|  | **Possible (3)** | 3 | 6 | | 12 | | 24 | | 48 |
|  | **Likely (4)** | 4 | 8 | | 16 | | 32 | | 64 |
|  | **Almost certain (5)** | 5 | 10 | | 20 | | 40 | | 80 |
| **Risk score= L x S** | | <6 | | 6 - 12 | | 13 - 32 | | >32 | |
| **Risk level** | | Low risk | | Medium risk | | High risk | | Very high | |
